# SAR image matching based on rotation-invariant description

**DOI:** 10.1038/s41598-023-41592-6

**Published:** 2023-09-04

**Authors:** Yunhao Chang, Qing Xu, Xin Xiong, Guowang Jin, Huitai Hou, Dan Man

**Affiliations:** 1 Institute of Geospatial Information, Information Engineering University, Zhengzhou, 450001 China; 2 Henan College of Surveying and Mapping, Zhengzhou, 450000 China

**Keywords:** Geomorphology, Core processes

## Abstract

The utilization of scale invariant feature transform algorithm in synthetic-aperture radar images (SAR–SIFT) to match image features may lead to principal orientation assignments of descriptors being affected by speckle noise, thereby diminishing accuracy. In this study, we propose using the Fourier histogram of oriented ratio gradient (Fourier HORG) descriptor for robust matching of SAR images. This method is based on the SAR–SIFT algorithm framework. During feature description, the rotation-invariant Fourier HORG descriptor is established by performing Fourier analysis on the ratio gradient in the polar coordinate system, whereby the principal orientation assignment process is avoided and the robustness of SAR image registration improved. A matching experiment was conducted involving four sets of SAR image pairs, and the results demonstrated that our method exhibited higher accuracy and robustness compared to image matching based on the Fourier histogram of oriented gradient (Fourier HOG) descriptor and the SAR–SIFT algorithm, thus confirming the effectiveness of our proposed method.

## Introduction

Synthetic aperture radar (SAR) is an active remote sensing system that is capable of obtaining high-precision images, regardless of independent of weather conditions. Thus, it is a valuable tool for a range of applications, including military reconnaissance, emergency relief, and deformation monitoring^1^. Image matching in SAR refers to the identification of identical points between the SAR images acquired under different conditions; this process is a fundamental step in various applications, such as image registration, mosaic creation, and change detection^2^. In contrast to the central projection method conventionally used in optical image acquisition, SAR images use slant range projection, resulting in distinctive geometric features such as shadow, layover, and foreshortening^3^. However, owing to random interference from electromagnetic wave signals, SAR images could be corrupted by speckle noise, resulting in blurry images^4^, thereby making it difficult to conduct automatic SAR image matching.

Image matching methods can be divided into grayscale- and feature-based methods. Grayscale-based methods are generally inefficient and sensitive to the geometric differences in the images. Therefore, relatively few relevant studies have been conducted using SAR images, and most existing literature on SAR image matching has employed feature-based methods. Although typical feature matching methods such as scale-invariant feature transform (SIFT)^5^ and speeded up robust features (SURF)^6^ perform well at matching optical images, their performance with SAR images is not as good. Several methods have been considered to achieve robust SAR image matching. For instance, Wang et al.^7^ proposed an algorithm based on a bilateral filter with SIFT, skipping the finest scale and using the dual-matching strategy to match the SAR images. Wang et al.^8^ proposed an adapted anisotropic Gaussian SIFT algorithm and adopted the dominant orientation consistency property to improve the matching stability. Dellinger et al.^9^ proposed the SAR-SIFT algorithm, using the ratio of exponentially weighted averages (ROEWA)^10^ to calculate the ratio gradient, which effectively reduced the influence of speckle noise in SAR images. In view of the superior performance of the SAR-SIFT algorithm for SAR image matching, several scholars have conducted advanced research in this area \* MERGEFORMAT 23. For example, Zou et al.^11^ introduced a new gradient definition and constructed a new scale space by making the registration algorithm suitable for polarimetric synthetic aperture radar (PolSAR) images. Pan et al.^12^ proposed a method with an improved SAR-SIFT algorithm based on kernel entropy component analysis (KECA). Ma et al.^13^ proposed a novel image matching method based on phase congruency (PC) and spatial constraint, which combine the advantages of PC and SAR-SIFT. Zhu et al.^14^ proposed a novel image matching method, using SAR-SIFT and R-SIFT to detect corner points and texture points, respectively, and using arborescence network matching for feature matching. Wang et al.^15^ proposed a uniform SAR-SIFT algorithm with optimal feature selection based on a Voronoi diagram, which optimized the spatial distribution of feature matches. Paul et al.^16–18^ proposed various improved SAR-SIFT algorithms, such as M UR-SIFT and I-SAR-SIFT, including novel feature extraction algorithms, descriptor construction methods, and matching algorithms. In another development, Yu et al.^19^ proposed embedding a rolling guidance filter into scale space construction.

To achieve image invariance to rotation in SAR image matching using the SAR-SIFT algorithm, it is necessary to assign one or more principal orientation to each feature. Owing to the speckle in SAR images, the accuracy of the principal orientation assignment of SAR-SIFT descriptors is usually inadequate, which leads to a decrease in the matching performance of the algorithm. To obtain rotation-invariant descriptors, Liu et al.^20^ proposed the rotation-invariant HOG descriptor using Fourier analysis. This descriptor has been used by several researchers, e.g., Wu et al.^21^ applied it to geospatial object detection of remote sensing imagery, Patel et al.^22^ used it for human action recognition, and Dong et al.^23^ used it to conduct ship detection. These studies showed that the Fourier HOG descriptor can be used effectively for object detection in optical images; however, few studies have been conducted on its use in image matching.

The Fourier HOG descriptor achieves rotation invariance without assigning the principal orientation, and the addition of relevant concepts to the SAR-SIFT algorithm could improve the matching performance. This study proposed an improved SAR-SIFT algorithm based on a Fourier histogram of oriented ratio gradient (Fourier HORG) descriptor to achieve robust SAR image matching. Based on SAR-SIFT, Fourier analysis of the ratio gradient in the polar coordinate system was used to obtain the rotation-invariant descriptor, avoiding poor matching owing to inaccurate principal orientation assignment.

## SAR image matching based on the Fourier HORG descriptor

**Figure 1:**
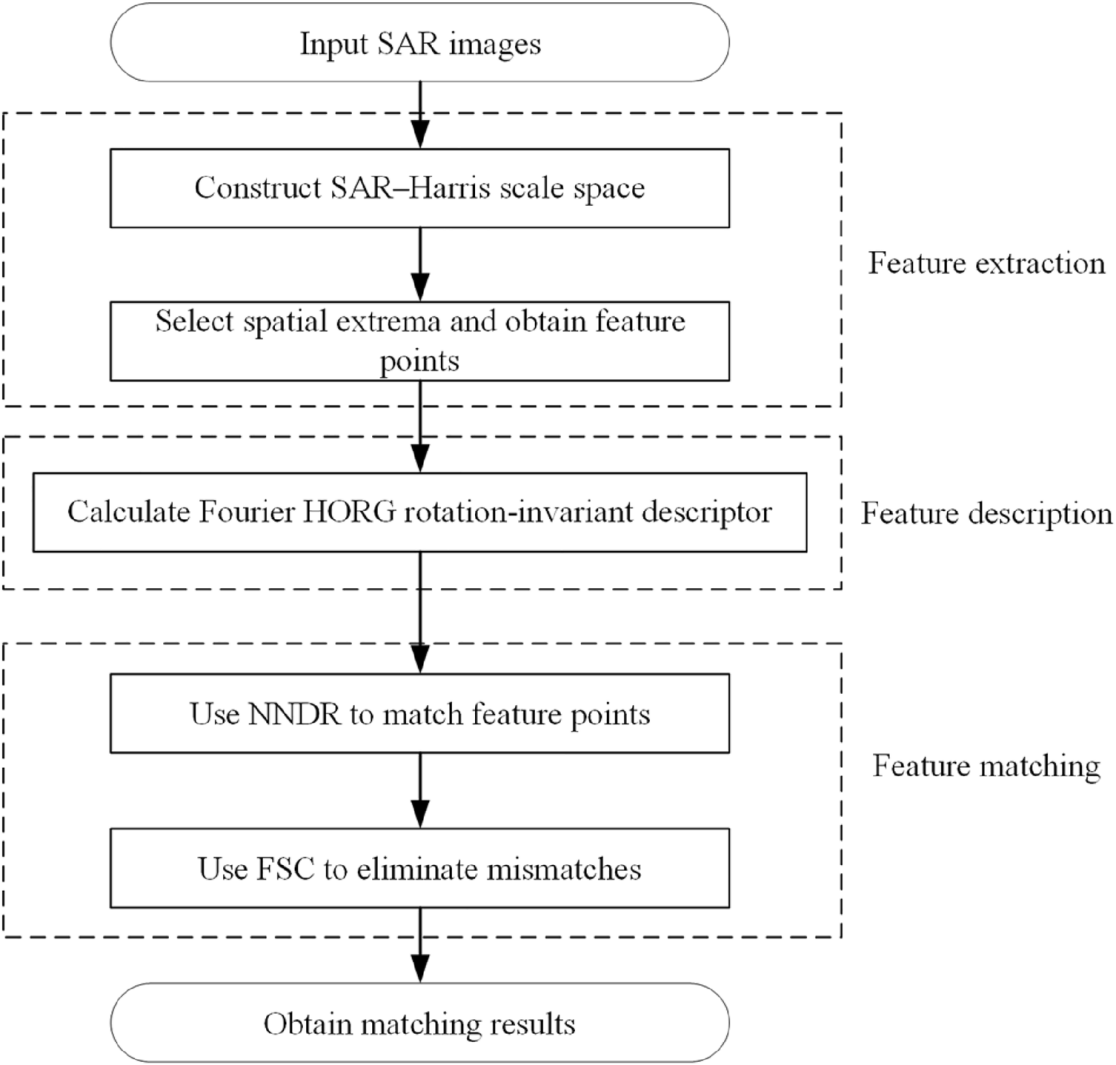
SAR image matching process based on Fourier HORG descriptor.

This study introduced the Fourier HORG descriptor to the SAR–SIFT algorithm to conduct SAR image matching. The process is shown in Fig. 1. We used the SAR–Harris algorithm to construct the scale space and extract the feature points of the SAR image. Subsequently, we used the Fourier HORG descriptor to describe the extracted feature points. Finally, we used the nearest neighbor distance ratio (NNDR)^24^ algorithm to match the feature points and the fast sample consensus (FSC) algorithm^25^ to eliminate mismatches, thereby obtaining the final matching result.

### SAR-Harris feature extraction

The SAR-SIFT algorithm uses the multi-scale SAR–Harris function to extract feature points, and is calculated as follows:

1 $$\begin{aligned} C_{\alpha } & = {\mathcal{G}}_{{\sqrt 2 \cdot \alpha }} *\left[ {\begin{array}{*{20}l} {\left( {G_{{x,\alpha }} } \right)^{2} } \hfill & {\left( {G_{{x,\alpha }} } \right) \cdot \left( {G_{{y,\alpha }} } \right)} \hfill \\ {\left( {G_{{x,\alpha }} } \right) \cdot \left( {G_{{y,\alpha }} } \right)} \hfill & {\left( {G_{{y,\alpha }} } \right)^{2} } \hfill \\ \end{array} } \right] \\ R_{\alpha } & = det\left( {C_{\alpha } } \right) - d \cdot tr\left( {C_{\alpha } } \right)^{2} \\ \end{aligned}$$

where $d=0.04$ is the constant, ${G}_{x,\alpha }$ and ${G}_{y,\alpha }$ are the horizontal and vertical gradients calculated using the ROEWA operator, respectively, and $\alpha$ is the parameter for calculating the local mean index weight. The ratio gradients ${G}_{x,\alpha }$ and ${G}_{y,\alpha }$ can be calculated as:

2 $$\begin{aligned} G_{x,\alpha } & = \log \left( {\frac{{\mathop \sum \nolimits_{i = - R}^{R} \mathop \sum \nolimits_{j = 1}^{R} I\left( {x + i,y + j} \right)e^{{ - \frac{\left| i \right| + \left| j \right|}{\alpha }}} }}{{\mathop \sum \nolimits_{i = - R}^{R} \mathop \sum \nolimits_{j = - R}^{ - 1} I\left( {x + i,y + j} \right)e^{{ - \frac{\left| i \right| + \left| j \right|}{\alpha }}} }}} \right) \\ G_{y,\alpha } & = \log \left( {\frac{{\mathop \sum \nolimits_{i = 1}^{R} \mathop \sum \nolimits_{j = - R}^{R} I\left( {x + i,y + j} \right)e^{{ - \frac{\left| i \right| + \left| j \right|}{\alpha }}} }}{{\mathop \sum \nolimits_{i = - R}^{ - 1} \mathop \sum \nolimits_{j = - R}^{R} I\left( {x + i,y + j} \right)e^{{ - \frac{\left| i \right| + \left| j \right|}{\alpha }}} }}} \right) \\ \end{aligned}$$

where $I$ is the SAR image, $\alpha$ is smoothing parameter, and $R=2\alpha$ is half the size of the processing window. Equation (1) extracts feature points at different scales ${\alpha }_{m}={\alpha }_{0}\cdot {c}^{m}$, wherein ${\alpha }_{0}$ is the scale of the initial layer and $m$ is the magnitude. Local extrema are selected as feature points at each scale.

### Fourier HORG feature description

For extracting descriptors, the SAR–SIFT algorithm utilizes a histogram of the gradient orientation within the local neighborhood of a feature to extract the principal orientation. One or more principal orientations can be calculated per feature point. By assigning the principal orientation, the SAR-SIFT descriptor can be rotation invariant. Nevertheless, owing to speckle in SAR images, the principal orientation assignment of SAR-SIFT descriptors is usually not sufficient, which leads to a decrease in the matching performance of the algorithm.

To avoid imprecise principal orientation assignment and improve the matching performance of the SAR-SIFT algorithm, we designed the Fourier HORG rotation-invariant descriptor and incorporated it into the SAR-SIFT algorithm framework. The Fourier HORG descriptor is an improvement of the Fourier HOG descriptor. Ratio gradients are used to calculate HOG and perform Fourier analysis at polar coordinates.

For ${\varvec{I}}\left(x,y\right)$ of an image, if $\left(x,y\right)$ represents the location of a certain pixel, the gradient orientation distribution function of the pixel is expressed as follows:

3 $$h\left( \varphi \right) = {\varvec{D}}\left( {x,y} \right)\delta \left( {\varphi - \theta \left( {{\varvec{D}}\left( {x,y} \right)} \right)} \right)$$

where ${\varvec{D}}\left(x,y\right)$ is the gradient of $\left(x,y\right)$, $\Vert {\varvec{D}}\left(x,y\right)\Vert$ and $\theta \left({\varvec{D}}\left(x,y\right)\right)$ are the gradient magnitude and orientation, respectively, and $\varphi$ is the angle of the gradient in polar coordinates.

The distribution function $h$ can be expanded using a Fourier series, and the coefficient of the Fourier series is as derived follows:

4 $$h\left( \varphi \right) = \mathop \sum \limits_{m = - \infty }^{\infty } f_{m} \left( {x,y} \right)e^{im\varphi }$$

The coefficient ${f}_{m}$ is calculated as follows:

5 $$f_{m} = \frac{1}{2\pi }\mathop \smallint \limits_{0}^{2\pi } h\left( \varphi \right)e^{ - im\varphi } d\varphi = {\varvec{D}}\left( {x,y} \right)e^{{ - im\theta \left( {{\varvec{D}}\left( {x,y} \right)} \right)}}$$

where $m$ is an integer, and $\left|m\right|\le T$. $T$ is the maximum frequency. To smooth the orientation function and improve the robustness of the descriptor, let $T=4$ 19. The coefficient ${f}_{m}$ encodes part of the information in $h$, and is used as an expression of the feature of the gradient.

To generate HORG features, spatial aggregation and local normalization must be performed on the gradient orientation distribution function. Convolution can be used to achieve these two processes. If ${\widehat{F}}_{m}$ is the intensive calculation of the Fourier coefficient ${f}_{m}$ and is the ${\varvec{D}}$ gradient field, then the $m$-th component ${\widetilde{F}}_{m}$ of the HORG field is

6 $$\tilde{F}_{m} = \frac{{\hat{F}_{m} *K_{1} }}{{\sqrt {{\varvec{D}}^{2} *K_{2} } }}$$

where ${K}_{1}$ is the spatial aggregation convolution kernel, and ${K}_{2}$ is the local normalization convolution kernel. According to Liu et al.^20^, the rotation order of ${\widetilde{F}}_{m}$ is $m$, and $\overline{{\widetilde{F} }_{m}}\cdot {\widetilde{F}}_{m}$ is a rotation-invariant feature.

To calculate rotation-invariant features and construct rotation-invariant descriptors for a larger spatial range, region descriptors must be generated based on the HORG field. As polar coordinates can separate the angular part from the radial part, which is rotation invariant, the polar coordinate system can be incorporated into the process. Sampling radially along the polar coordinate system, the two-dimensional basis function of the region descriptor is calculated as follows:

7 $$\begin{aligned} U_{j,k} \left( {r,\varphi } \right) & = {\Lambda }\left( {r - r_{j} ,\sigma } \right)e^{ik\varphi } \\ {\Lambda }\left( {x,\sigma } \right) & = max\left( {\frac{\sigma - x}{\sigma },0} \right) \\ \end{aligned}$$

where $k$ is the rotation order of the convolution kernel, $r$ is the radial value in polar coordinates, ${r}_{j}$ is the $j$-th radial scale of the basis function, $\sigma$ is half of the triangular function $\Lambda$. According to Li et al.^20^, ${U}_{j,k}\left(r,\varphi \right)*{\widetilde{F}}_{m}$ has the rotation order $k-m$ and, if this is multiplied with its complex conjugate $\overline{{U }_{j,k}\left(r,\varphi \right)*{\widetilde{F}}_{m}}$, and the case where the rotation order is equal is extracted, the following is obtained:

8 $$\begin{gathered} \overline{{\left( {U_{{j_{1} ,k_{1} }} *\tilde{F}_{{m_{1} }} } \right)}} \left( {U_{{j_{2} ,k_{2} }} *\tilde{F}_{{m_{2} }} } \right) \hfill \\ \forall k_{1} - m_{1} = k_{2} - m_{2} \hfill \\ \end{gathered}$$

Equation (8) is the Fourier HORG descriptor, which is rotation invariant.

### Feature matching and mismatch elimination

After the features are extracted and described, it is necessary to match them in the two images. In this study, we used the NNDR algorithm^24^ for feature matching and the FSC algorithm^25^ to enhance the matching results.

The NNDR is a classic feature matching algorithm comprising two steps, namely calculation of the Euclidean distance between the descriptor in one image and the other image to determine the nearest and second-nearest neighbors; thereafter, calculation of the ratio of the distance between the nearest and second-nearest neighbors and elimination of the point pairs with a ratio less than the threshold. The last remaining point pairs are the matched feature point pairs.

The FSC is an improved random sample consensus^26^ algorithm that quickly and robustly extracts effective matches from a set containing a large number of outliers and refines the results. The specific steps are as follows:

1. Select an appropriate mapping model between matching points, with n pairs of matching points required to establish the model;
2. Set a threshold and select a certain number of matching point pairs from the preliminary matching results as the sample data set;
3. Randomly select n pairs of matching points in the sample data set to be interior points;
4. Establish a mapping model based on these interior points;
5. Substitute all remaining matching points into the mapping model, with the point pairs conforming to the model being interior points and the non-conforming point pairs being the exterior points according to the set residual threshold;
6. Repeat steps 3–5 to the preset iteration threshold, and compare the number of interior points obtained by each model;
7. Finally, the correct match is the result with the largest number of interior points.

## Experiment

Four pairs of SAR images were used in a matching experiment conducted to verify the performance of the Fourier HORG descriptor. The basic information on the image pairs is shown in Table 1. The experimental data were obtained from the ALOS-PALSAR, Sentinel-1, TerraSAR-X, UAV and aircraft platforms, including the L, C, X, and Ku wave-bands, covering Zhengzhou, Luohe, Tianjin, Dengfeng, Taiyuan and Zhenjiang, respectively. Image pairs 1, 3 and 5 had rotation differences, image pair 3 had scale differences, image pair 4 had a UAV SAR image with considerable geometric distortion and image pair 5 was taken from opposite sides.

**Table 1:** Parameters of experimental SAR image pairs.

No	Platform	Waveband	Polarization method	Size (pixels)	Sample interval (m)	Date captured	Region
1	ALOS-PALSAR	L	HH	600 × 600	30 × 30	22 Sep 2008	Zhengzhou
	ALOS-PALSAR	L	HH	600 × 600	30 × 30	22 Dec 2008	
2	Sentinel-1	C	VV	800 × 800	10 × 10	6 May 2016	Luohe
	Sentinel-1	C	VH	800 × 800	10 × 10	23 May 2016	
3	TerraSAR-X	X	HH	500 × 500	20 × 20	18 Apr 2009	Tianjin
	ALOS-PALSAR	L	HH	800 × 800	12.5 × 12.5	7 May 2008	
4	UAV	Ku	HH	1000 × 1000	0.06 × 0.12	24 Apr 2021	Dengfeng
	UAV	Ku	HH	1000 × 1000	0.06 × 0.12	24 Apr 2021	
5	UAV	Ku	VV	1000 × 1000	0.5 × 0.5	28 Nov 2020	Taiyuan
	UAV	Ku	HH	1000 × 1000	0.5 × 0.5	28 Nov 2020	
6	Aircraft	Ku	HH	1000 × 1000	0.3 × 0.3	13 Apr 2021	Zhenjiang
	Aircraft	Ku	HH	1000 × 1000	0.3 × 0.3	15 Apr 2021	

**Figure 2:**
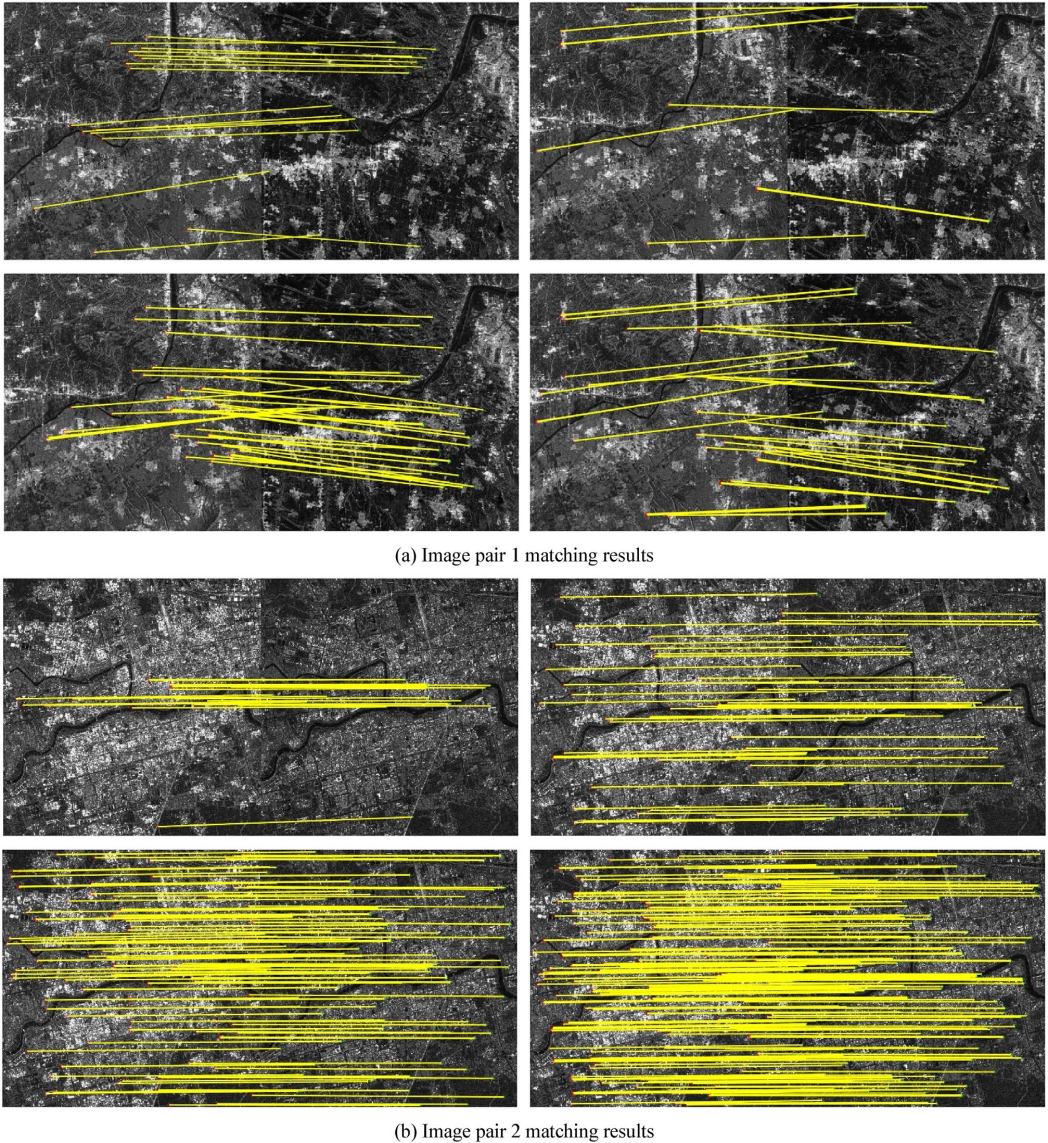

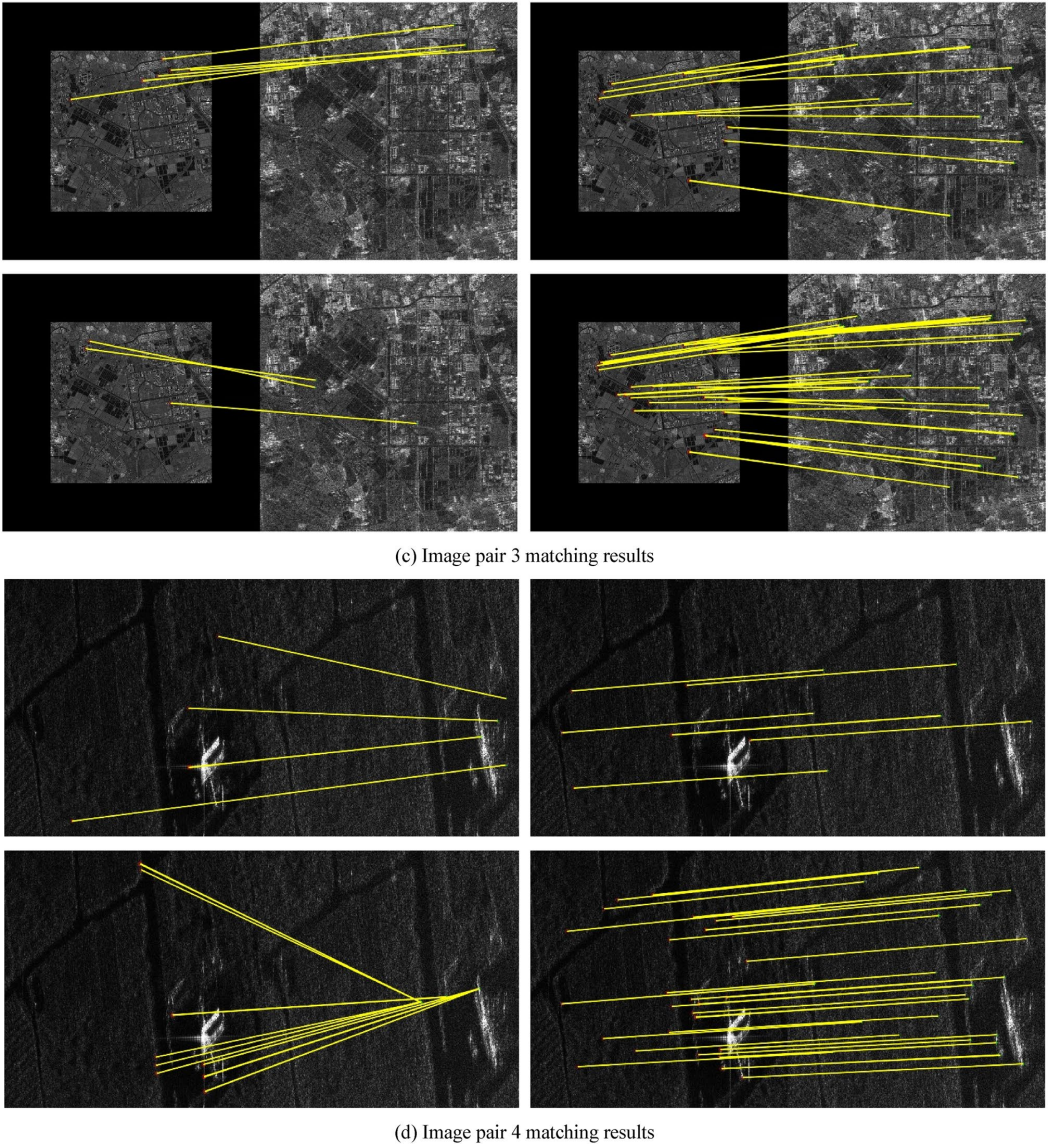

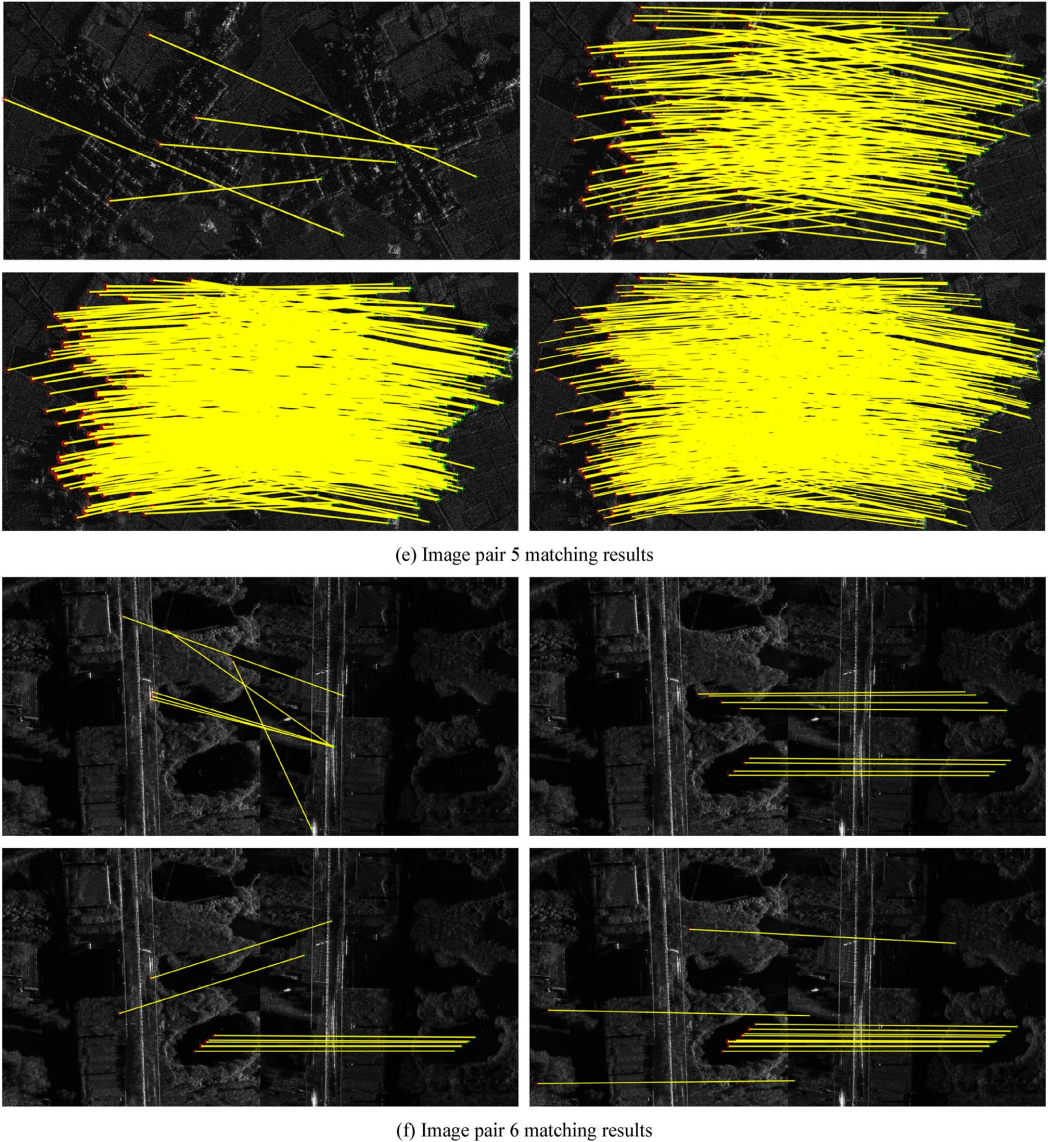
Matching results of three methods for SAR image pairs.

A comparison was conducted between the proposed Fourier HORG descriptor and the SIFT algorithm, and the SAR-SIFT descriptor and Fourier HOG descriptors. Except for the SIFT algorithm, the other descriptors use the SAR-Harris operator to extract the feature points (the parameter settings are available in Dellinger et al.^9^). Further, the NNDR method was used to match the descriptors, and the FSC operator was used to refine the matching results. The evaluation indicators in the experiment included factors such as the number of matching point pairs, matching accuracy, root mean square error of matching point pairs, and matching time. The number of matching points is the number of point pairs found by the FSC algorithm, matching accuracy is the number of correct matches as a ratio of the total number, and the method for determining correct matches was based on that of Xiong et al.^2^. The root mean square error is the root mean square of the coordinate residuals of the matching pair (unit pixels). The matching time is the operational time required for the matching calculations for each pair of images. We used the following computer configuration for the statistical time: Windows 10 21H1 × 64 operating system, Intel i7-6700HQ CPU, 16G RAM, and NVIDIA GTX980M GPU. The matching results are shown in Table 2 and Fig. 2.

**Table 2:** Comparison of match results.

Image pair	Method	Number of matching pairs	Accuracy (%)	RMSE	Matching time (s)
1	SIFT	14	85.7	4.71	**5.54**
	SAR-SIFT	10	80.0	2.02	53.62
	Fourier HOG	40	85.0	2.31	37.77
	Fourier HORG	**43**	**88.3**	**1.91**	40.77
2	SIFT	21	80.9	6.83	**9.91**
	SAR-SIFT	51	98.3	2.26	78.90
	Fourier HOG	136	84.6	2.24	56.75
	Fourier HORG	**273**	**99.3**	**1.89**	68.77
3	SIFT	8	87.5	2.98	**4.77**
	SAR-SIFT	13	92.3	1.83	28.54
	Fourier HOG	–	–	–	–
	Fourier HORG	**41**	**92.7**	**1.77**	52.12
4	SIFT	–	–	–	–
	SAR-SIFT	7	71.5	7.53	**84.95**
	Fourier HOG	–	–	–	–
	Fourier HORG	**37**	**89.2**	**2.08**	105.47
5	SIFT	–	–	–	–
	SAR-SIFT	235	98.3	1.70	160.78
	Fourier HOG	978	98.4	1.71	**73.38**
	Fourier HORG	**1499**	**98.7**	**1.69**	74.63
6	SIFT	–	–	–	–
	SAR-SIFT	8	50.0	9.34	109.84
	Fourier HOG	–	–	–	–
	Fourier HORG	**27**	**96.3**	**1.15**	**71.19**

“–” denotes failed matching attempt.

Significant values are given in Bold.

Table 2 shows that the Fourier HORG descriptor-based matching method had the largest number of matching point pairs and the highest accuracy rate, indicating that its superior performance. This result could be attributed to the Fourier HORG method using the ratio gradient to overcome the effect of speckle in the SAR images, which avoids the inaccurate principal orientation assignment process and directly constructs the rotation-invariant descriptor, which increases the rotation invariance of the descriptor. The only successful match of the SIFT algorithm was for image pairs 1–3, with the overall number of matches and the accuracy rate not being particularly high. This result was ascribed to the difference gradient used for feature matching, with the description being affected significantly by SAR image speckle noise that also affected the accuracy of the principal orientation assignment. Except for image pair 5, the SAR-SIFT algorithm achieved few matching point pairs and had poor accuracy. This result was ascribed to speckle reducing the accuracy of the principal orientation assignment and making it difficult to ensure the performance of the SAR-SIFT descriptor. The Fourier HOG descriptor-based method successfully matched only image pairs 1, 2 and 5, ascribed to the difference gradient of the image being extremely sensitive to speckle noise. Consequently, the Fourier HOG descriptor, which is based on the difference gradient, could not be applied to SAR images.

The results of the coordinates of matching point pairs obtained by the four methods in the control group indicated that both the method proposed in this study and the SAR-SIFT algorithm achieved the same four matching point pairs in image pair 1, 42 pairs in image pair 2, 232 pairs in image pair 5 and 4 pairs in image pair 6; however, identical matching point pairs were not achieved in image pairs 3 and 4. The matching point pairs obtained using SIFT and Fourier HOG differed from those obtained using the other two methods. This result showed that the smaller the rotation difference between the two images, the higher would be the point pair repeatability between the SAR-SIFT algorithm and the method proposed in this study. This indicated that if the feature extraction results were the same, the Fourier HORG descriptor had superior rotation invariance. In terms of root mean square error, the Fourier HORG descriptor had the highest matching accuracy, followed by the SAR-SIFT algorithm, further confirming the superior performance of the Fourier HORG descriptor. In terms of matching time, SIFT is an algorithm in the programming platform MATLAB® (MathWorks, Natick, Natick, Massachusetts, USA), which performed relatively well. The average matching time of the SAR-SIFT algorithm was 86.11 s and that of the Fourier HORG descriptor-based method was 68.83 s. Although both techniques yielded similar results, the performance of the Fourier HORG algorithm was marginally superior. The Fourier HORG descriptor-based method obtained more matching point pairs with higher efficiency, accuracy and precision and, overall, its performance was superior to that of the other three methods.

The matching results are shown in Fig. 2. In each set of four images, the top left, top right, bottom left, and bottom right images indicate the matching results of the SIFT algorithm, SAR-SIFT algorithm, Fourier HOG descriptor-based method, and Fourier HORG descriptor-based method, respectively. Both Fourier HORG descriptor-based method and SAR-SIFT algorithm successfully matched the 6 image pairs and obtained a certain number of matching points; however, the distribution of the matching point pairs obtained using the method introduced in this study was more uniform and had more quantities. The SIFT algorithm successfully matched image pairs 1–3, but its accuracy was lower, and the point distributions were not ideal. The Fourier HOG-based method successfully matched only image pairs 1, 2 and 5, and the matched results were not distributed uniformly.

**Figure 3:**
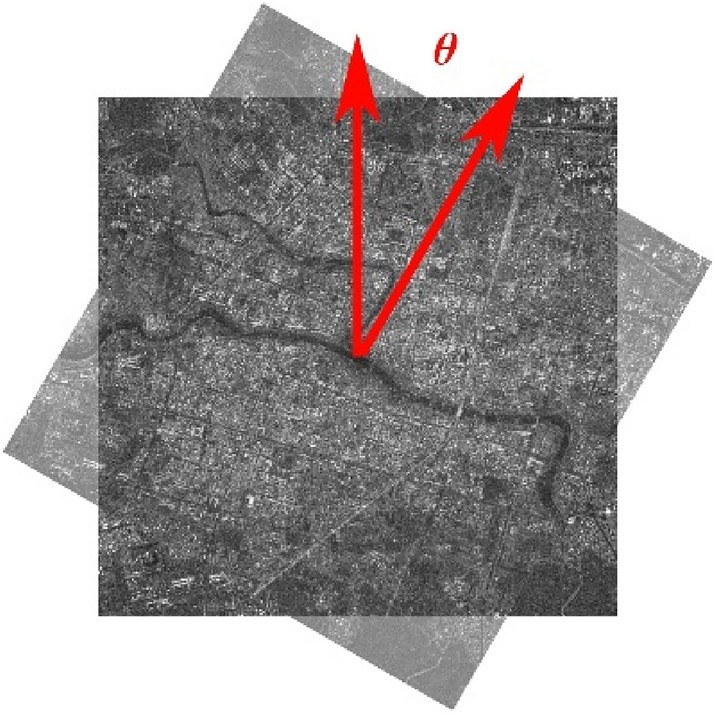
Rotation example of image pair 2.

**Figure 4:**
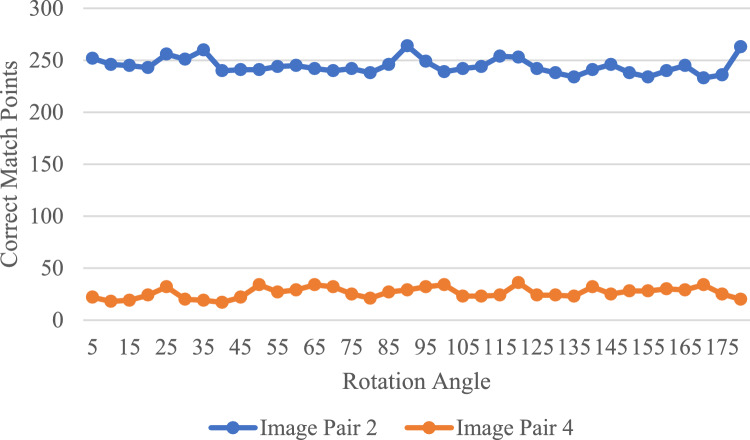
Number of correct matching points at different rotation angles.

To evaluate the rotation invariance of the Fourier HORG descriptor, we conducted an experiment using image pairs 2 and 4, with almost no difference being found between the two image pairs. For each pair of images, we first rotated the second image clockwise, as shown in Fig. 3 and, subsequently, used the Fourier HORG descriptor-based method to conduct matching with the first image. The rotation angle $\theta$ varied from 0° to 180°, with the matching experiment performed every 5°. Afterward, the number of correct matching points were counted (Fig. 4). We found that despite the number of correct matching points in the two pairs of images fluctuating slightly, they remained stable overall, with standard deviations of 7.62 and 5.19, respectively, confirming the good rotation invariance of the Fourier HORG descriptor (Supplementary file).

## Conclusion

This study proposed a robust matching method for SAR images based on Fourier HORG descriptors. Based on the SAR-SIFT algorithm framework, we used Fourier analysis of the ratio gradient in the polar coordinate system to obtain rotation-invariant Fourier HORG descriptors. The result of an experiment involving four sets of SAR images showed that the method proposed in this study achieved higher accuracy and robustness than the Fourier HOG descriptor-based image matching method and the SAR-SIFT algorithm, as well as the distribution was more uniform. The method performed well for both satellite and UAV images, which is a promising finding for future studies in fields such as image registration and adjustment computation. In the rotation-invariance experiment, the method proposed in this study performed well at each rotation angle, thereby confirming the rotation invariance of the descriptors. Nevertheless, the experimental results showed the limited computational efficiency of the Fourier HORG descriptor-based matching method. Therefore, future studies should focus on developing methods to enhance the efficiency of SAR image matching while also ensuring its robustness.

### Supplementary Information

Supplementary Information.

## Data Availability

All data generated or analysed during this study are included in this published article and its supplementary information files.
