# Supplementary figures and images for: SAR image matching based on rotation-invariant description
Source: Sci Rep. 2023 Sep 4;13:14510. doi: 10.1038/s41598-023-41592-6 (PMC10477315; doi:10.1038/s41598-023-41592-6)

## Raw Data

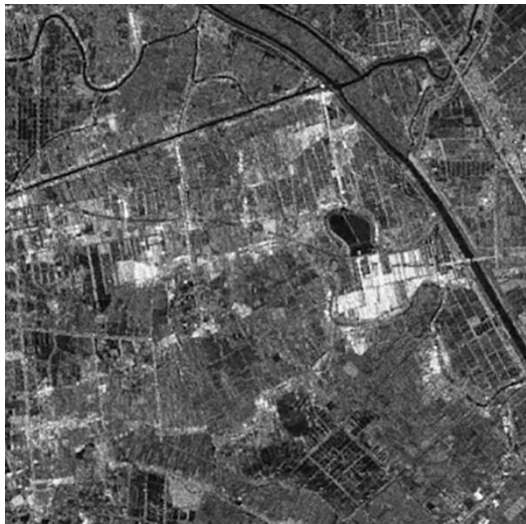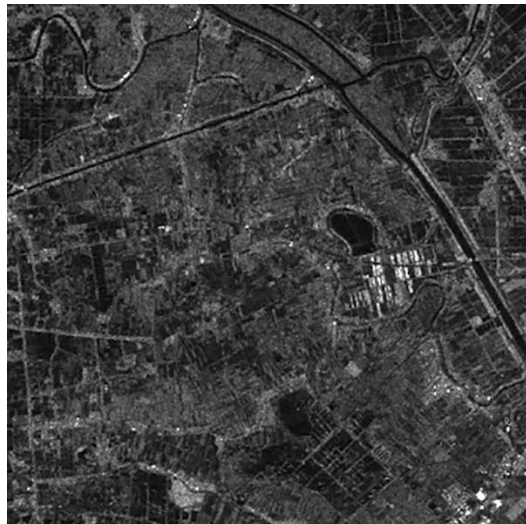

Image pair 1

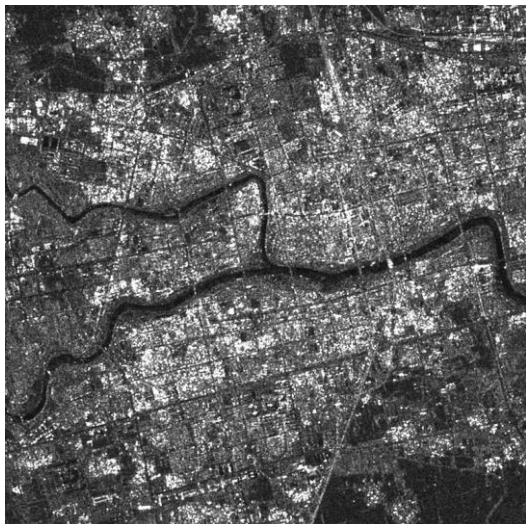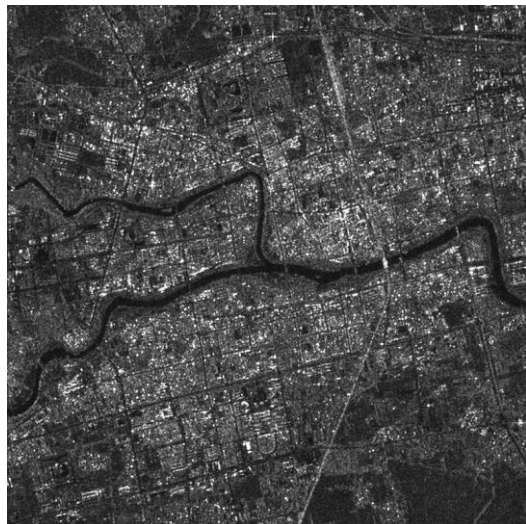

Image pair 2

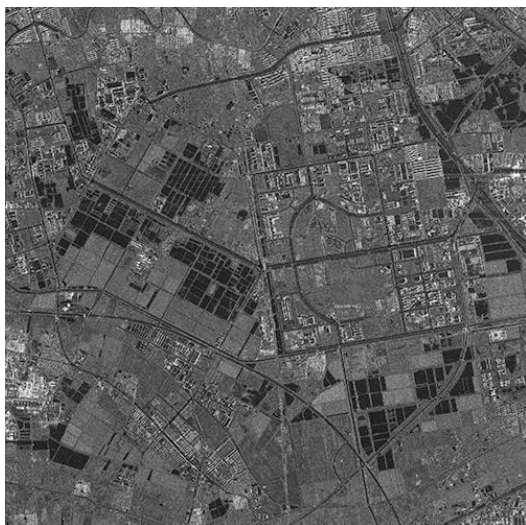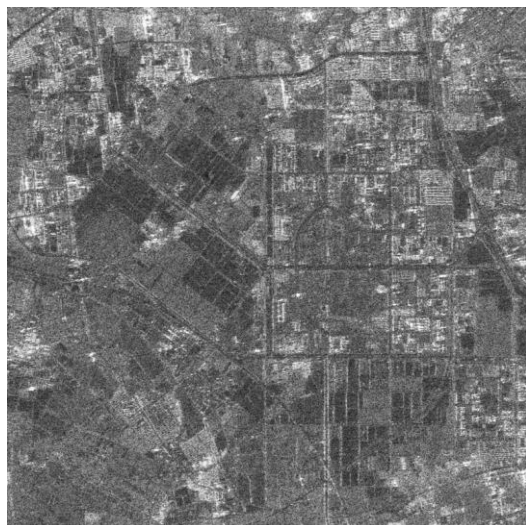

Image pair 3

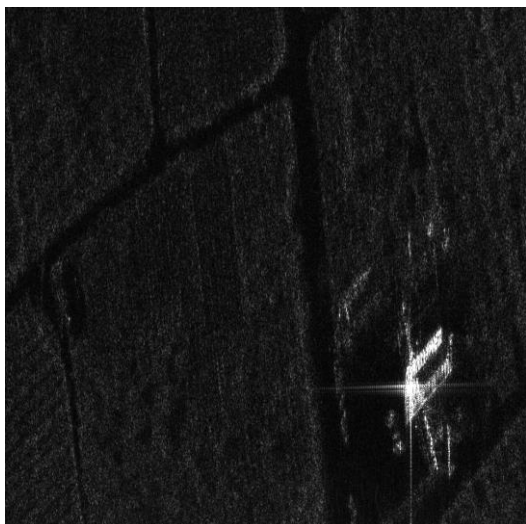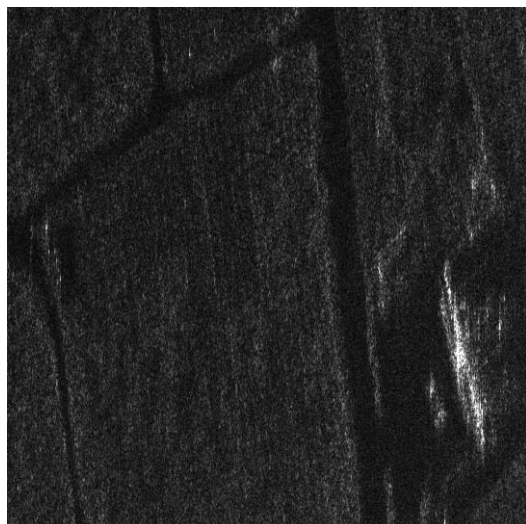

Image pair 4

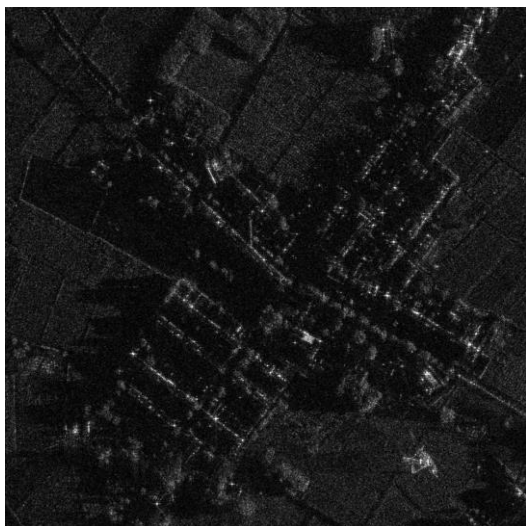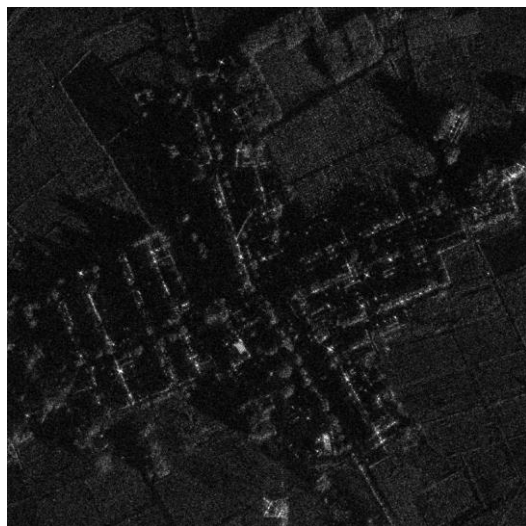

Image pair 5

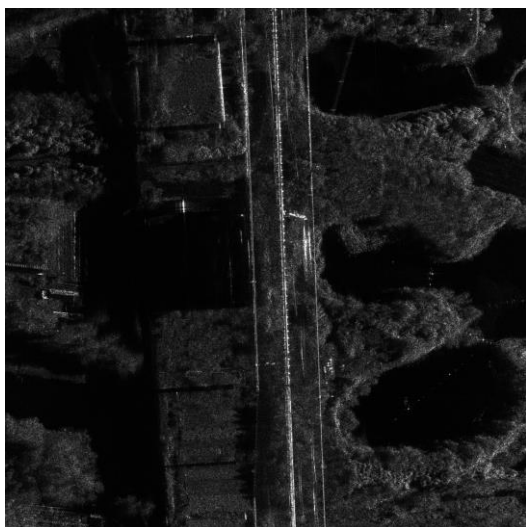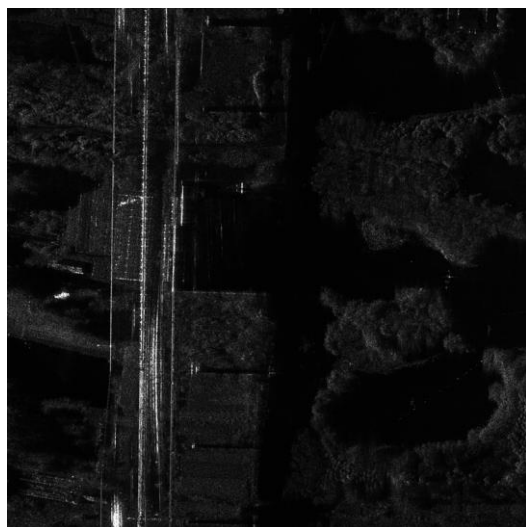

Image pair 6

Supplement: Supplementary file 1 — Supplementary Information. [file 41598_2023_41592_MOESM1_ESM.pdf]
